# Tumour-Derived sEVs Promote Triple-Negative Breast Cancer Progression Associated with HAVCR2 Upregulation in Macrophages

**DOI:** 10.32604/or.2026.079137

**Published:** 2026-07-16

**Authors:** Jia Liu, Binqian Wang, Yannan Jin, Wenquan Chen, Ruohan Shi, Weijia Wang, Xiaojing Zhang, Yi Tan, Zhongran Man, Bo Hu, Lisen Zhu, Biao Zhang, Chongchan Bao, Gongsheng Jin

**Affiliations:** 1 Department of Oncological Surgery, First Affiliated Hospital of Bengbu Medical University, Bengbu, China; 2 Anhui Province Key Laboratory of Basic and Translational Research of Inflammation-Related Diseases, Bengbu Medical University, 2600 Donghai Avenue, Bengbu, China; 3 Department of Hepatopancreatobiliary Surgery, the First Affiliated Hospital of Bengbu Medical University, Bengbu, China; 4 Department of Breast and Thyroid Surgery, Affiliated Hospital of Youjiang Medical University for Nationalities, Baise, China; 5 Key Laboratory of Molecular Pathology in Tumors of Guangxi Higher Education Institutions, Affiliated Hospital of Youjiang Medical University for Nationalities, Baise, China

**Keywords:** Breast cancer, triple-negative breast cancer, tumour microenvironment, HAVCR2, sEVs, M2-associated marker

## Abstract

**Backgrounds:** Triple-negative breast cancer (TNBC) is the most aggressive breast cancer subtype with a unique tumor microenvironment, and while Programmed cell death protein 1/Programmed cell death ligand 1 (PD-1/PD-L1) blockade represents a standard immunotherapy, most patients develop primary or acquired resistance, with few alternative immunotherapeutic targets currently available. Therefore, we aimed to identify potential immune checkpoint-related molecules involved in TNBC-macrophage crosstalk, clarify the underlying molecular mechanism mediated by small extracellular vesicles (sEVs), and provide a theoretical basis for the future development of novel immunotherapeutic targets against TNBC. **Methods:** Single-cell RNA-sequencing (scRNA-seq) datasets for various breast cancer subtypes were used. Pseudotime trajectory, cell‒cell communication and Tumor Immune Estimation Resource 2.0 (TIMER2) analyses were conducted to characterize the tumour microenvironment (TME). Immunochemistry and immunofluorescence were used to confirm the results of the above analyses. Single-nucleus RNA sequencing (snRNA-seq) was conducted on three pairs of TNBC tumour and adjacent normal tissues. The functions of tumour-associated macrophages (TAMs) and sEVs in TNBC metastasis were explored by Western blotting, flow cytometry and cell-based experiments. **Results:** A total of nearly 60,000 high-quality single cells were subjected to scRNA-seq analysis, from which seven major cell types were identified. An overall increase in immune cell proportion was observed in TNBC compared with other subtypes, with the immune cell fraction in TNBC tissues being ~1.8-fold higher than that in luminal A/HER2+ subtypes (*p* < 0.001). Cell‒cell communication analysis indicated that TNBC cells mainly interact with macrophages. Interestingly, HAVCR2, an immune checkpoint, is expressed mainly in macrophages in the TNBC TME. HAVCR2 is associated with macrophage pseudotime progression in TNBC, which was validated by immunofluorescence staining. Moreover, analysis of The Cancer Genome Atlas (TCGA) bulk RNA-seq data revealed that HAVCR2 expression is significantly correlated with M2-like macrophage gene signatures and computationally inferred macrophage infiltration levels in TNBC, and this tissue-level transcriptional correlation is associated with poor patient prognosis. Notably, bulk RNA-seq data cannot define discrete cell subsets, and the identification of HAVCR2+ M2 macrophage subsets was independently validated by scRNA-seq and snRNA-seq at the single-cell level. Furthermore, treatment with TNBC-derived sEVs is associated with concurrent increases in the expression of HAVCR2 and M2-associated markers (CD163, CD206) in macrophages. These findings reflect a correlative association rather than a demonstrated causal or regulatory relationship between HAVCR2 and M2-associated marker upregulation. **Conclusion:** sEVs derived from TNBC cells are associated with the upregulation of M2-associated markers and concomitant HAVCR2 upregulation in macrophages, both of which correlate with TNBC progression and metastasis. We propose that HAVCR2 may serve as a candidate prognostic marker associated with M2-like macrophage features in TNBC, and these foundational *in vitro* findings from Human acute monocytic leukemia cell line (THP-1) macrophages warrant further validation in primary human monocyte-derived macrophages and *in vivo* TNBC models.

## 1 Introduction

Breast cancer is a heterogeneous disease encompassing multiple subtypes with distinct histological and molecular characteristics [1]. Subtyping is primarily based on immunohistochemical expression of the estrogen receptor (ER), progesterone receptor (PR), and human epidermal growth factor receptor 2 (HER2) [2]. Luminal and triple-negative breast cancer (TNBC) are the most common cancers among women worldwide [3]. TNBC is defined by the absence of ER, PR and HER2 expression [4]. The Luminal subgroup consists of luminal A (ER+/PR+, HER2−) and luminal B (ER+/PR−, HER2−) subtypes, while HER2-positive breast cancer is characterized by an ER^−^/PR^−^, HER2^+^ phenotype [2]. The prognosis is highly variable among these subtypes, with substantial prognostic disparities across groups [5].

The tumour microenvironment (TME) contains cancer cells, stromal cells, and various immune cell subsets [6]. The TME plays a fundamental role in the tumorigenesis and development of breast cancer by affecting processes such as proliferation, angiogenesis, immune system inhibition and drug resistance [7,8,9]. Unlike that of other subtypes, the TME of TNBC contains many tumour-infiltrating immune cells with prognostic value, suggesting that immunotherapies targeting immune cells have the potential to prolong the survival of TNBC patients [10]. While immune checkpoint inhibitors (ICIs) prolong survival for patients with many solid tumour types, anti-PD-L1/PD-1 antibodies, the currently most commonly used ICIs in TNBC treatment, yield limited clinical responses. Approximately 15%–20% of TNBC patients obtain clinical benefits from single-agent ICI therapy, whereas most patients exhibit inherent or acquired resistance [11].

Macrophages are major components of the TME [12]. Macrophages residing within the TME display distinct molecular phenotypes in response to diverse tumour-derived stimuli. Classically activated M1 macrophages, characterized by proinflammatory cytokine secretion, confer antitumour activities, while macrophages with upregulated M2-associated markers are correlated with cancer progression via secretion of pro-tumor cytokines [13,14,15]. Macrophage infiltration in most solid tumour types is associated with poor clinical outcomes [16]. In particular, breast cancer tissues exhibit relatively higher macrophage infiltration levels relative to other malignant tumours [17].

Research on the correlation between the TME and tumour progression has demonstrated that sEVs are pivotal in intercellular communication between BC cells and the premetastatic niche [18,19]. As bilayer membrane vesicles ranging from 30 to 150 nm in size, sEVs carry nucleic acids, proteins and lipids, serving as essential signaling messengers within the TME [20]. Tumour-derived sEVs deliver pro-tumorigenic cues to stromal cells and immune cells, thereby inducing immune suppression, facilitating angiogenesis and remodeling the extracellular matrix. Meanwhile, these sEVs can traffic to distant organs to form a pre-metastatic niche and lay the foundation for tumour distant metastasis [21]. Exosomal non-coding RNAs, particularly microRNAs, act as core bioactive molecules regulating crosstalk between tumour cells and immune cells in the TME; however, their precise mechanisms governing immune checkpoint expression in macrophages remain poorly defined [22,23,24]. 

This study aimed to investigate the potential associations of TNBC-derived sEVs with macrophage polarization and the expression of the immune checkpoint molecule HAVCR2 within the TME, as well as to assess the potential of HAVCR2 as a potential prognostic biomarker and immunotherapeutic target for TNBC. We hypothesized that TNBC-derived sEVs are correlated with M2-like macrophage polarization and upregulate HAVCR2 expression, and these phenotypic alterations are linked to TNBC progression and metastasis. These observations may generate testable hypotheses regarding HAVCR2 as a novel immunotherapeutic candidate for TNBC treatment.

## 2 Materials and Methods

### 2.1 Data Resources

Breast cancer scRNA-seq data, including the GSE195861 and GSE180286 datasets, were obtained from the Gene Expression Omnibus (GEO) [25,26]. The overall dataset contains information on primary tumour tissue samples from 13 patients with four breast cancer subtypes. The Cancer Genome Atlas (TCGA) RNA-Seq data of primary breast tumours were downloaded from the Genomic Data Commons Data Portal (https://gdc.cancer.gov). TNBC subtype classification and survival information were obtained from a previous study [27]. Basal-subtype breast cancer negative for ER, PR and HER2 was considered TNBC.

### 2.2 scRNA-Seq Analysis

Single-cell clustering and annotation were performed via the “Seurat” package (version 4.3.0) in R software. The cluster similarity spectrum (CSS) method was used to integrate samples. The CellChat package (version 1.1.3) in R software was used to evaluate cell‒cell communication interactions in the four breast cancer subtypes. The monocle package (version 2.28.0) in R software was used to illustrate the cell state transition in macrophages of TNBCs. Prior to integration, strict quality control was performed on raw scRNA-seq data to remove low-quality cells and reduce technical noise: ① cells with unique molecular identifier (UMI) counts < 200 or >60,000 were excluded; ② cells with gene counts < 200 were excluded; ③ cells with mitochondrial gene expression > 15% of total expression were excluded; ④ low-expression genes detected in <3 cells were filtered out. After QC, a total of 59,872 high-quality cells were retained from 13 patient samples for subsequent analysis, with no significant batch effects observed in UMI/gene counts across patient/subtype groups. Batch correction was performed using the Harmony algorithm (a standard method for scRNA-seq batch effect removal) to eliminate technical variation across patient samples and sequencing batches, with “sample” and “sequencing batch” set as batch covariates [28]. After batch correction, the Cluster Similarity Spectrum (CSS) method was used for cell type integration and clustering, with the following key parameters: resolution = 0.8, k-nearest neighbor (kNN) = 20, and principal component analysis (PCA) dimensionality reduction to 30 principal components (PCs) (based on elbow plot analysis of PCA variance explained). Cell type annotation was performed using marker gene-based supervised classification, with canonical marker genes for each cell type (e.g., EPCAM for epithelial cells, CD68/CSF1R for macrophages, CD3D/CD4 for T cells, MS4A1 for B cells) validated against the PanglaoDB and CellMarker databases. Reanalysis of scRNA-seq data was conducted to quantify the transcriptional expression of canonical M1-associated marker genes in TAMs of different breast cancer subtypes.

### 2.3 Immune Infiltration Analysis

TIMER is a comprehensive resource for the systematic analysis of immune infiltrates across human cancers. Immune infiltration data for TCGA cancer types are available from the website (http://timer.cistrome.org/). Data from the Tumor Immune Estimation Resource (TIMER), Molecular Characterization of Peripheral Cells Counter (MCPCOUNTER) and Estimating the Proportion of Immune and Cancer cell types (EPIC) methods were used in this study [29].

### 2.4 Immunofluorescence Assay

Multiplex staining of FFPE tissue was performed using the PANO 4-plex IHC kit (Panovue, Cat# 0004100100; Panovue, Beijing, P. R. China) according to the manufacturer’s instructions. Briefly, FFPE tissue slides were first deparaffinized and then incubated with primary antibodies against CD68 (CST, Cat# 26042), MRC1 (CST, Cat# 24595S), and HAVCR2 (CST, Cat# 45208S), followed by horseradish peroxidase-conjugated secondary antibody incubation and tyramide signal amplification. The slides were microwave heat-treated after each tyramide signal amplification operation. Nuclei were stained with DAPI (MedChemExpress, HY-D0814) after all the antigens above had been labelled. The immunofluorescence images were captured via a Carl Zeiss LSM900 microscope (Oberkhorn, Germany) with fluorescence spectra at 20-nm wavelength intervals from 420 to 720 nm and identical exposure times; For each slide, 5 fields enriched in immune cells were selected for imaging. All the scans for each slide were then superimposed to obtain a single image. The multilayer images were imported into ImageJ (version 1.52p, National Institutes of Health, USA) for quantitative image analysis.

### 2.5 Human Tissue and Single-Nucleus RNA Sequencing (snRNA-seq) Sample Acquisition

The project was performed in compliance with the guidelines outlined in the Declaration of Helsinki. Studies using human tissues were approved by the Institutional Ethical Review Boards of Bengbu Medical University (approval number: Runco Batch No. 316 [2024], 10 July 2024) and performed in accordance with the principles of Declaration of Helsinki. These samples were obtained from patients who had been admitted to the First Affiliated Hospital of Bengbu Medical University and had received a diagnosis of TNBC on the basis of pathological examination. After the patients signed informed consent forms, we collected surgical tumour tissues and adjacent tumour tissues from three patients and sent them to a sequencing company for single-cell sequencing.

### 2.6 Nucleus Preparation

For single-nucleus RNA sequencing library preparation, nuclei were extracted from frozen tissue specimens using a commercial Nucleus Isolation Kit (SHBIO, Cat# 52009-10; Shanghai SHBIO Co., Ltd., Shanghai, P. R. China). RNase inhibitors (Sigma-Aldrich, Cat# 3335399001; Germany) were added to all buffers immediately before use. Tissue fragments were finely minced on ice, transferred to 5 mL centrifuge tubes with lysis solution. After 3 min of incubation on ice, the crude lysate was passed a 40 μm cell filter (Sigma BAH136800040). Nuclear yield and integrity were initially assessed using a SeekMate Tinitan Fluorescence Cell Counter (SeekGene M002C) with AO/PI dual staining. After staining with 0.4% trypan blue (Sangon Biotech E607320-0001), the nuclei were observed under a 40× microscope (Jiangnan Novel Optics XD-202). Samples with >90% intact nuclei and negligible cytoplasmic contamination were selected for downstream processing.

### 2.7 Single-Nuclei RNA Sequencing Library Construction and Sequencing

For single-nucleus transcriptomic profiling, snRNA-seq libraries were generated using the SeekOne^®^ Digital Droplet Single Cell 3’ Library Preparation Kit (SeekGene, Catalogue No. K00202; China). In brief, a defined number of purified cell nuclei were mixed with reverse transcription reagent and loaded into the designated wells of a SeekOne^®^ DD Chip S3. Barcoded hydrogel beads (BHBs) and droplet partitioning oil were then added separately to the corresponding chip compartments. After microfluidic emulsion formation, reverse transcription was carried out at 42°C for 90 min, and the reaction was inactivated at 85°C for 5 min. Next, cDNA was extracted from disrupted emulsions, purified, and amplified via PCR. The amplified cDNA was then processed through a series of enzymatic reactions including fragmentation, end repair, dA-tailing, and sequencing adapter ligation. Finally, indexed PCR amplification was subsequently performed to amplify the DNA representing the 3’ polyA region of the expressed genes, which also contained a cell barcode and a unique molecular index. The resulting libraries were cleaned up with VAHTS DNA Clean Beads (Vazyme, Cat# N411-01; Vazyme Biotech Co., Ltd., China) and analysed with a Qubit (Thermo Fisher Scientific, Cat# Q33226; USA) and a Bio-Fragment Analyzer (Bioptic, Qsep400; BiOptic Inc., China). High-quality libraries were finally sequenced on an Illumina NovaSeq 6000 using the PE150 sequencing strategy.

### 2.8 Statistical Analysis

All statistical analyses were performed via GraphPad Prism 9 (GraphPad Software, San Diego, California, USA) and R software 4.1.0 (R Foundation for Statistical Computing, Vienna, Austria); *p* < 0.05 was considered statistically significant. Pearson’s R value was obtained in the correlation analysis. For survival analysis, patients were classified into two groups on the basis of the expression level of HAVCR2. We then referred to previously described methods and evaluated the results via Kaplan–Meier survival analysis [30]. *p* < 0.05 was considered to indicate statistical significance.

### 2.9 Immunohistochemistry (IHC)

FFPE tissue blocks from 15 TNBC patients (divided into three groups according to progression-free survival: short-term recurrence group [PFS ≤ 3 years, *n* = 5], intermediate recurrence group [3 years < PFS < 5 years, *n* = 5], good prognosis group [PFS ≥ 5 years, *n* = 5]) who had undergone resection for breast cancer were used for IHC. These patients were divided into three groups according to progression-free survival (PFS): patients with PFS ≤ 3 years were included in the short-term recurrence group, patients with 3 years < PFS < 5 years were included in the intermediate recurrence group, and patients with PFS ≥ 5 years were included in the good prognosis group. The samples were subjected to anti-HAVCR2 (CST, Cat# 45208S, USA) and anti-CD163 antibodies (CST, Cat# 93498S, USA). All the staining processes were performed using a DAB substrate kit (Elabscience^®^, E-IR-R101, China). The H-score method was used for quantification: intensity score (0 = none, 1 = weak, 2 = moderate, 3 = strong) was assigned based on average staining intensity of positive cells, and overall score was calculated as 3 × % strongly stained + 2 × % moderately stained + 1 × % weakly stained. All the markers were scored within the tumour margin. Five random fields of view (100 × magnification) were evaluated under a Leica DM3000 light microscope (Leica Microsystems). The IHC score was determined by two independent pathologists who were blinded to the patients’ clinical features and outcomes.

### 2.10 Cell Lines, Culture Conditions and M0 Macrophage Induction

MDA-MB-231, BT-549, MCF7, MCF10a and THP-1 cells were purchased from the Chinese Academy of Sciences. All cell lines were authenticated by short tandem repeat (STR) profiling and tested negative for mycoplasma contamination by Nanning Rising Biotechnology Co., Ltd. before use. THP-1 cells were cultured in RPMI 1640 medium (KeygenBioTECH, KGL1501-500) supplemented with 10% foetal bovine serum (FBS; Gibco, Life Technologies, New Zealand), 1% penicillin-streptomycin (Dowobio, Shanghai, China, DW0023), and 0.05 mM β-mercaptoethanol at 37°C with 5% CO_2_. To induce M0 macrophage differentiation, THP-1 cells were treated with 100 ng/mL PMA for 24 h, after which adherent cells were confirmed as M0 macrophages.

### 2.11 Coculture Experiments

To analyse the influence of different cell interactions, a coculture system was established using Transwell chambers (0.4 μm, Corning, New York, NY, USA). The macrophages were placed in the lower chamber, and the MDA-MB-231 BT-549, MCF7 and MCF10A cells were placed in the upper chamber. This setup was used because the smaller polycarbonate membrane does not allow cells to migrate into each other.

### 2.12 Total Protein Extraction and Western Blotting (WB)

After coculture with breast cancer cells (MDA-MB-231, MCF7, or MCF10A), M0 macrophages were lysed by RIPA lysis buffer (Servies, Wuxi, China) supplemented with a protease inhibitor (MedChemExpress, NJ, USA; HY-K0010) for protein extraction. Protein concentrations were quantified using an Abbkine BCA protein assay kit (KTD3001, China). Equal amounts of protein were loaded onto 10% sodium dodecyl sulphate‒polyacrylamide gels and separated via electrophoresis. The separated proteins were subsequently transferred to polyvinylidene fluoride (PVDF) membranes. The PVDF membranes were then incubated in 5% skim milk for approximately 1 h at room temperature. The membranes were incubated with primary antibodies (HAVCR2, Abcam, Cambridge, UK, 1:1000; β-actin, Nature Biosciences, A94160, China, 1:2000; GAPDH, Affinity Biosciences, USA, 1:3000; CD163, Proteintech, Wuhan, China, 1:1000; CD9, Zenbio, R380441, 1:1000; CD63, Zenbio, 340219, 1:1000; TSG101, Proteintech, Wuhan, China, 1:1000) at 4°C overnight. The membranes were subsequently incubated with the secondary antibody at room temperature for at least 1 h the next day. All the WB experiments were repeated at least three times. Protein expression was detected via a Bio-Rad imaging system and ImageJ software.

### 2.13 Flow Cytometry

To detect macrophage markers, the cells were labelled with BV421 Mouse Anti-Human CD68 (Y1/82A) and PE Mouse Anti-Human CD206 (19.2) (BD Pharmingen, USA). Specifically, the pretreated macrophages were digested with Accutase Cell Detachment Solution (BD Pharmingen, USA) for digestion, and digestion was terminated with medium containing serum. The precipitate was then resuspended in 100 μL of PBS; 5 μL of Human BD Fc Block (Fc1.3216) (BD Pharmingen, USA) was added, and the sample was finally mixed and incubated for 10 min to block the Fc receptor. Then, the rupture of the cell membranes was induced by fixation/permeabilization for 45 min. After washing with PBS, the cells were resuspended in 300 μL of PBS with 5 μL of anti-CD68 antibody and 20 μL of anti-CD206 antibody for 45 min for visualization of the intracellular expression of these proteins. The percentage of CD68^+^/CD163^+^ macrophages was quantified via a BD FACSCantoBrea flow cytometer (BD Biosciences, Becton, Dickinson and Company, USA).

### 2.14 Isolation and Identification of sEVs

sEVs were extracted by sequential ultracentrifugation. Briefly, the culture medium was replaced with medium containing 10% EV-depleted FBS. Then, the cell culture medium was collected and centrifuged at 300× *g* for 10 min, 2000× *g* for 15 min, and finally, at 12,000× *g* for 30 min to remove cell debris. After filtration through 0.22-μm filters (Jet Biofil, FPE2024030), the supernatant was ultracentrifuged at 100,000× *g* (Optima XPN-100) for 2 h. The sEV pellets were washed in PBS and ultracentrifuged at 100,000× *g* for another 2 h. All the samples were resuspended in PBS for further analyses. The morphology of the sEV particles was characterized via transmission electron microscopy (JEM-1400FLASH Japan). WB was employed to analyze sEV protein markers.

### 2.15 Wound Healing Assay

Wound healing assays were used to evaluate the migration ability of breast cancer cells cocultured with macrophages, and normal breast cancer cells were used as the control group. The cells were cultured in a 6-well plate. When confluency reached 95%, wound tracks were created by scraping the cell monolayer with sterile pipette tips. The detached cells were gently removed by washing three times with PBS. The cells were subsequently cultured in standard conditions and photographed at 0 h, 24 h and 48 h under a microscope. The scratched are quantified with ImageJ software.

### 2.16 Cell Migration and Invasion Assays

The migration ability was measured via a Transwell migration assay using cell culture inserts. Cocultured breast cancer cells (1 × 10^4^ cells per insert) in 200 μL of medium without FBS were seeded in the upper chamber, and 600 μL of medium containing 20% FBS was added to the lower chamber. Following an incubation period ranging from 24 to 96 h, the cells were fixed with 4% paraformaldehyde and subsequently stained with 0.1% crystal violet (SL7081, Coolaber, China). The stained cells were imaged under a Shunyu optical microscope (Ningbo Sunny Instrument Co., Ltd., China). 

### 2.17 Cell Counting Kit-8 (CCK-8) Assay

The viability of both cocultured BT-549 and MDA-MB-231 cells was assessed via the CCK-8 assay. 2 × 10^3^ cells per well were seeded in 96-well plates and incubated for 24 h, 48 h, 72 h, or 96 h under standard conditions. CCK-8 solution (Biolight, CCK001) was added to each well, and the plate was incubated for 1–4 h. The absorbance of each well was measured using a microplate reader.

## 3 Results

### 3.1 Large-Scale Integration of scRNA-Seq Profiling Data for Patients with Four Breast Cancer Subtypes

**Figure 1:**
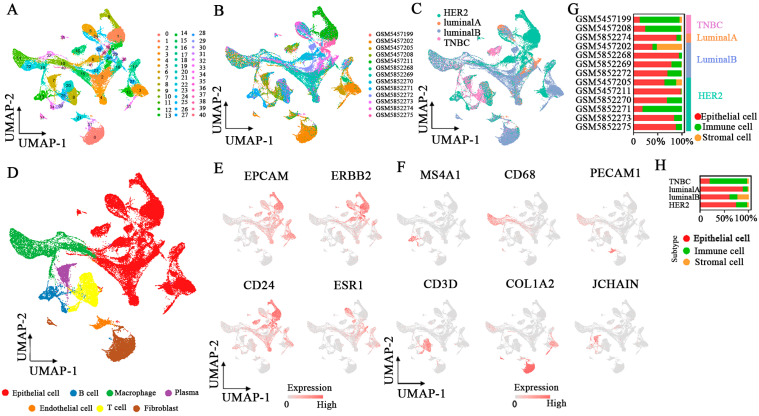
Large-scale integration of scRNA-seq profiling data from patients with four breast cancer subtypes. (**A**) Clustering with dimensional reduction based on the Seurat package and the CSS method. (**B**,**C**) Clustering of patients (**B**) and breast cancer subtypes (**C**) based on (**A**). (**D**) Clustering of major cell types based on (**A**). (**E**,**F**) Clustering based on the expression of marker genes of epithelial cells (**E**) and immune and stromal cells (**F**) based on (**A**). (**G**,**H**) Proportion of epithelial, immune and stromal cells in each patient (**G**) and breast cancer subtype (**C**). CSS: Cluster Similarity Spectrum.

To elucidate the cellular landscape of different subtypes of breast cancer, we used GEO 10x Genomics scRNA-seq datasets (GSE180286 and GSE195861) containing data on primary tumour tissue samples from 13 patients [25]. Nearly 60,000 high-quality cells were obtained for subsequent analysis. To elucidate the cellular landscape of breast cancer tumours, we applied the CSS method for cell type identification (Fig. 1A–C). With marker-based annotations, seven major cell types, including epithelial cells, B cells, macrophages, plasma cells, endothelial cells, T cells and fibroblasts, were identified in the breast cancer samples obtained for scRNA-seq (Fig. 1D–F). We found that the cell types remained well separated, clustered together, and were mixed between batches. Similar to previous studies, some the proportions of epithelial cells were highly variable among patients and subtypes [31]. Interestingly, compared with other subtypes, an overall increase in the proportion of immune cells was observed in TNBC, which is consistent with the findings of a previous study reporting that this subtype comprises many tumour-infiltrating immune cells (Fig. 1G,H). Prior to integration, strict quality control (QC) was performed to remove low-quality cells (UMI < 200/>60,000, mitochondrial gene expression > 15%), retaining 59,872 high-quality cells from 13 patient samples. Batch effects across patient and sequencing batches were eliminated using the Harmony algorithm, and the CSS method was used for cell type integration and clustering (30 PCs, kNN = 20, resolution = 0.8). With marker-based annotations (validated against PanglaoDB/CellMarker), seven major cell types were identified in the breast cancer samples.

### 3.2 Global Communication among Multiple Cell Types in Each Breast Cancer Subtype

**Figure 2:**
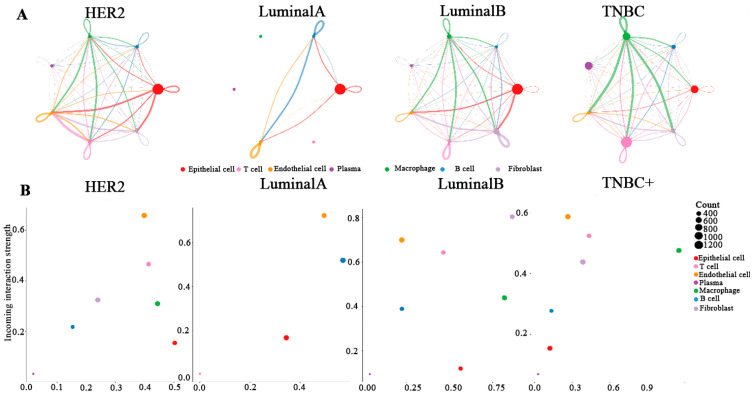
Global communication among multiple cell types in each breast cancer subtype. (**A**) Circos plot showing the inferred intercellular communication network across major cell types in each breast cancer subtype. (**B**) Dot plot showing the outgoing and incoming interaction strengths based on (**A**).

Single-cell data analysis can reveal putative cell-extrinsic interactions, as this approach allows integration of ligand and receptor information [32]. Next, we explored the putative interactions among seven major cell types in each breast cancer subtype (Fig. 2). Luminal A samples had the fewest cell–cell interactions. Epithelial cells were involved a relatively greater number of cell–cell interactions in the HER2 and luminal B subtypes. Fibroblasts were involved in the most cell–cell interactions in the luminal B subtype. Macrophages were observed to have more cell–cell interactions in the HER2 and luminal B subtypes and particularly the TNBC subtype.

### 3.3 Landscape of IC Expression in Four Breast Cancer Subtype Patients

**Figure 3:**
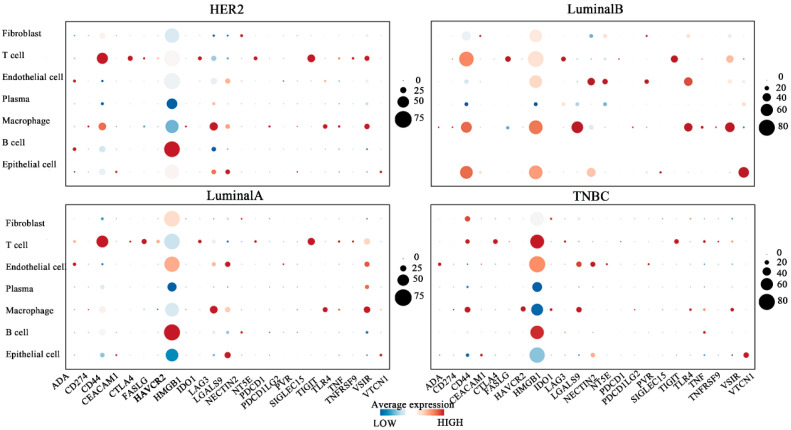
Landscape of IC expression in four breast cancer subtypes. Dot plot showing the IC expression levels (average expression) and proportion of expressing cells (dot size) among major cell types in each breast cancer subtype. IC: immune checkpoint.

ICs are frequently expressed in the TME, promote immune evasion and regulate antitumour immune responses. We assessed the expression levels and percentages of cells expressing 23 ICs in this breast cancer scRNA-seq dataset [33]. The results revealed that CD44 is more highly expressed in the T cells of luminal B patients than in those of patients with other subtypes (Fig. 3). HMGB1 is expressed in almost every major cell type of the four breast cancer subtypes and is expressed at the highest level in B cells. CTLA4 and FASLG are expressed mainly in the T cells of the three subtypes. HAVCR2 is expressed in the macrophages of four subtypes of breast cancer and is expressed at higher levels in TNBC than in other subtypes. LAG3, PDCD1 and TIGIT are expressed mainly in the T cells of most breast cancer subtypes. LGALS9 is expressed mainly in the macrophages of four breast cancer subtypes and is also expressed in the endothelial cells of TNBC patients. NECTIN2 is expressed in almost every major cell type of the four breast cancer subtypes. VSR is expressed mainly in the T cells and macrophages of the four breast cancer subtypes. These results suggest that intercell types/subtypes are conserved and that the expression of these ICs is heterogeneous in breast cancer patients. Reanalysis of this scRNA-seq dataset further revealed that canonical M1-associated marker genes (TNF, IL1B, CXCL9, CXCL10, NOS2, CD86, HLA-DRA) in TNBC TAMs were significantly lower than those in macrophages of luminal A/HER2+ breast cancer subtypes (*p* < 0.05), and were barely detectable in HAVCR2+ macrophages with upregulated M2-associated markers. This *in vivo* transcriptomic evidence indirectly supports that TNBC-associated macrophages lack M1 activation, complementing *in vitro* M2 marker data.

### 3.4 HAVCR2 Expression Correlates with Upregulation of M2-Associated Markers in TNBC

**Figure 4:**
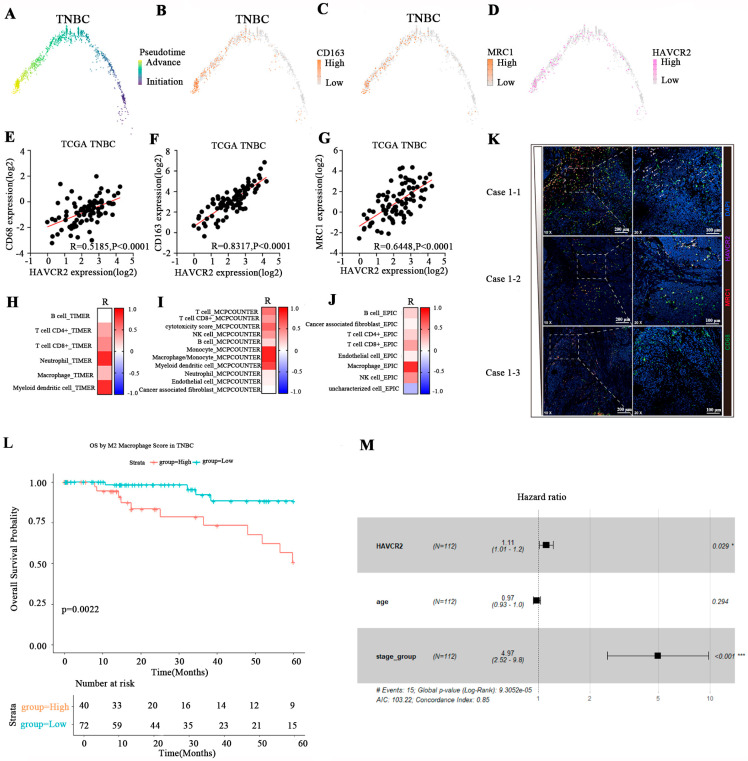
HAVCR2 expression is associated with macrophages with upregulated M2-associated markers in TNBC. (**A**–**D**) The developmental trajectory of macrophages in TNBC was inferred by Monocle using a semisupervised learning model; the trajectory is colour-coded by pseudotime (**A**), CD163 expression (**B**), MRC1 expression (**C**) and HAVCR2 expression (**D**). (**E**–**G**) Correlations between HAVCR2 expression and the transcript levels of M2-like macrophage markers CD163 (**E**), MRC1 (**F**) and pan-macrophage marker CD68 (**G**) in TCGA TNBC bulk RNA-seq data. (**H**–**J**) Heatmap showing the correlation between HAVCR2 expression and immune infiltration levels of TNBCs based on TIMER (**H**), MCPCOUNTER (**I**) and EPIC (**J**) data. (**K**) Multicolour immunofluorescence images of TNBC samples showing subpopulations of macrophages co-expressing HAVCR2 and M2-associated marker MRC1. Scale bar: 200 μm (main image), 100 μm (inset). (**L**,**M**) The relationship between computationally inferred M2-like macrophage enrichment scores, HAVCR2 gene expression and patient prognosis in the TCGA TNBC bulk RNA-seq cohort. TNBC: Triple-negative breast cancer; CD: Cluster of differentiation; HAVCR2: Hepatitis A virus cellular receptor 2; MRC1: Mannose receptor C-type 1; TIMER: Tumor Immune Estimation Resource; MCPCOUNTER: Microenvironment Cell Populations-counter. *p < 0.05; ***p < 0.001.

According to the abovementioned findings, an overall increase in the proportion of immune cells was observed in TNBC. Additionally, macrophages, particularly those of the TNBC subtype, have been shown to be involved in more cell-cell interactions. Immunotherapeutic approaches targeting immune cells have the potential to improve survival in patients with TNBC. Thus, we performed pseudotime developmental trajectory analysis (Fig. 4A). We found that the M2-like macrophage marker genes CD163 and MRC1 were highly enriched at the end of the trajectory axis (Fig. 4B,C), indicating that macrophages with upregulated M2-associated markers are present at the end of triple-negative breast cancer differentiation. Interestingly, HAVCR2 expression was found to be associated with macrophage pseudotime progression (Fig. 4D). Moreover, our analysis of TCGA bulk RNA-seq data from TNBC tissues revealed that HAVCR2 expression was significantly correlated with the transcript levels of macrophage pan-marker CD68 and M2-like macrophage markers CD163 and MRC1 (Fig. 4E–G). Correlation analysis further revealed that HAVCR2 expression had a stronger positive correlation with the transcript levels of M2-like macrophage markers (CD163: R = 0.8317, *p* < 0.0001; MRC1: R = 0.6448, *p* < 0.0001) than with those of total macrophage marker CD68 (R = 0.4265, *p* < 0.001), suggesting that HAVCR2 expression is closely associated with M2-like macrophage transcriptional signatures rather than general macrophage infiltration at the tissue level in the TNBC TME. 

Immune infiltration analysis revealed that HAVCR2 expression was positively correlated with the proportions of immune cells, particularly macrophages, but not with those of stromal cells (Fig. 4H–J). These results indicate that HAVCR2^+^ macrophages with upregulated M2-associated markers are likely present in TNBC and likely play an important role in tumour regulation. To further study the relationship among M2-associated marker enrichment, HAVCR2 expression and triple-negative breast cancer progression, we used TCGA bulk RNA-seq data from 112 TNBC patients to deeply explore the relationship among computationally inferred M2-associated marker enrichment scores, HAVCR2 gene expression levels and patient prognosis (Fig. 4K,L). Survival analysis showed that patients with highly enriched M2-associated markers had significantly lower overall survival rates than those in the low-enrichment groups. The pvalue of the log-rank test was 0.0022, indicating that the large number of macrophages with upregulated M2-associated markers were closely linked to the adverse prognosis of TNBC patients, which demonstrated that macrophages with upregulated M2-associated markers played a cancer-promoting role in the tumor microenvironment of TNBC and promoted tumor growth and metastasis. 

The HAVCR2 gene expression analysis suggests that its expression is closely related to prognosis. The hazard ratio (HR) in the high-expression group was 1.11, the 95% confidence interval was (1.01–1.2), and the *p* value was 0.029, indicating that the high expression of HAVCR2 is significantly related to the poor prognosis of TNBC patients (Fig. 4M). These findings suggest that HAVCR2 expression is closely correlated with TNBC progression, and its high expression may serve as a prognostic indicator for poor outcomes in TNBC patients. For further verification, we collected triple-negative breast cancer samples and performed multicolor immunofluorescence staining. Quantitative analysis revealed a high percentage of CD68^+^ macrophages co-expressed HAVCR2 and MRC1, confirming that HAVCR2 was co-expressed with macrophage marker CD68 and M2-associated marker MRC1 (Fig. 4K). These results again confirmed the presence of subpopulations of macrophages co-expressing HAVCR2 and M2-associated markers in triple-negative breast cancer, which were related to the adverse prognosis of the patients.

### 3.5 Analysis of snRNA-Seq Data Confirmed That a High Proportion of Macrophage Subsets in TNBC Are Characterized by High Expression of HAVCR2

**Figure 5:**
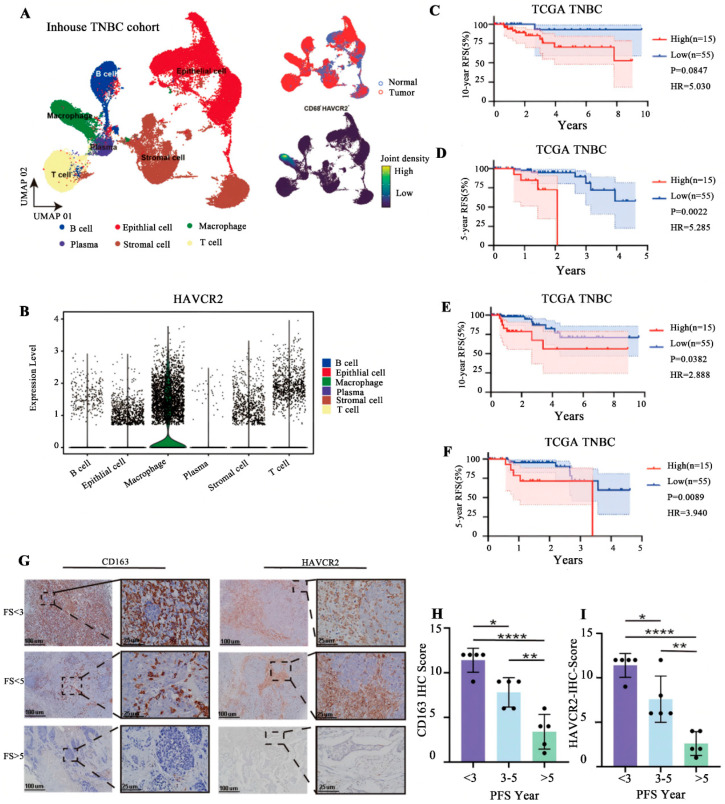
A high proportion of macrophages exhibit high expression of HAVCR2 in TNBC, and HAVCR2 expression is associated with poor survival in TNBC patients. (**A**) Clustering with dimensionality reduction based on the Seurat package and the CSS method, the cell-type distribution for the tumour samples and the normal samples, and the localization of CD68^+^ HAVCR2+ cells among all cell types. (**B**) HAVCR2 was found to be highly expressed in macrophages via quantitative analysis of global cellular expression. (**C**–**F**) Kaplan-Meier plots showing the OS and RFS of TCGA TNBC patients grouped according to HAVCR2 expression. The red lines represent patients with high HAVCR2 expression, and the blue lines represent patients with low HAVCR2 expression. (**G**–**I**) Representative IHC images showing HAVCR2 staining in TNBC samples from patients with different clinical outcomes. Scale bar: 100 μm (main image), 25 μm (inset). All data were analyzed by using unpaired Student’s *t*-tests and are shown as the means ± SD. *p < 0.05; **p < 0.01; ****p < 0.0001. TNBC: Triple-negative breast cancer; CSS: Cluster Similarity Spectrum; HAVCR2: Hepatitis A virus cellular receptor 2; OS: Overall Survival; RFS: Recurrence-free Survival; TCGA: The Cancer Genome Atlas.

To determine the cell types present in breast tumours, we applied CSS methods for cell type identification. We analysed snRNA-seq data from three pairs of TNBC tumour tissues and adjacent normal tissues. Marker-based annotation identified six major cell types in this breast cancer scRNA-seq dataset, including epithelial cells, B cells, macrophages, plasma cells, T cells, and stromal cells. Further analysis of the cell distribution in tumour and normal samples, as well as the localization of CD68^+^HAVCR2^+^ cells across all cell populations, revealed that most CD68^+^HAVCR2^+^ cells colocalized with macrophages. Quantitative analysis of HAVCR2 expression was performed for all cells (Fig. 5A,B) and revealed that HAVCR2 was highly expressed in macrophages. These findings validate our previous public dataset analyses, confirming a high proportion of HAVCR2-high macrophages in TNBC. Of note, this in-house snRNA-seq analysis was based on only three TNBC patients. Given TNBC heterogeneity, these data serve as supportive evidence consistent with public scRNA-seq findings, rather than providing definitive conclusions regarding HAVCR2-high macrophage subsets in TNBC.

### 3.6 HAVCR2 Expression Is Associated with Poor Survival in TNBC Patients

We analysed the TCGA BRCA bulk RNA-seq cohort and found that high HAVCR2 expression, which correlates with M2-like macrophage enrichment scores, was associated with poor 5-year and 10-year OS and RFS in TNBC patients (Fig. 5C–F). IHC staining revealed that HAVCR2 and CD163 expression was upregulated in patients with adverse clinical outcomes (Fig. 5G–I). To clarify the independent prognostic value of HAVCR2, we performed multivariate Cox proportional hazards regression analysis on the TCGA TNBC cohort (*n* = 112), adjusting for established clinical prognostic factors including clinical stage, tumor grade, age and M2 macrophage enrichment score. The results showed that high HAVCR2 expression remained an independent prognostic factor for poor overall survival (OS) in TNBC patients (HR = 1.09, 95% CI: 1.01–1.18, *p* = 0.032), while M2 macrophage enrichment score, clinical stage and age showed no significant independent prognostic correlation in this model, indicating that HAVCR2 exerts prognostic effects independent of macrophage abundance and conventional clinical factors.

### 3.7 TNBC Cells Correlate with Upregulated M2-Associated Markers and HAVCR2 In Vitro

**Figure 6:**
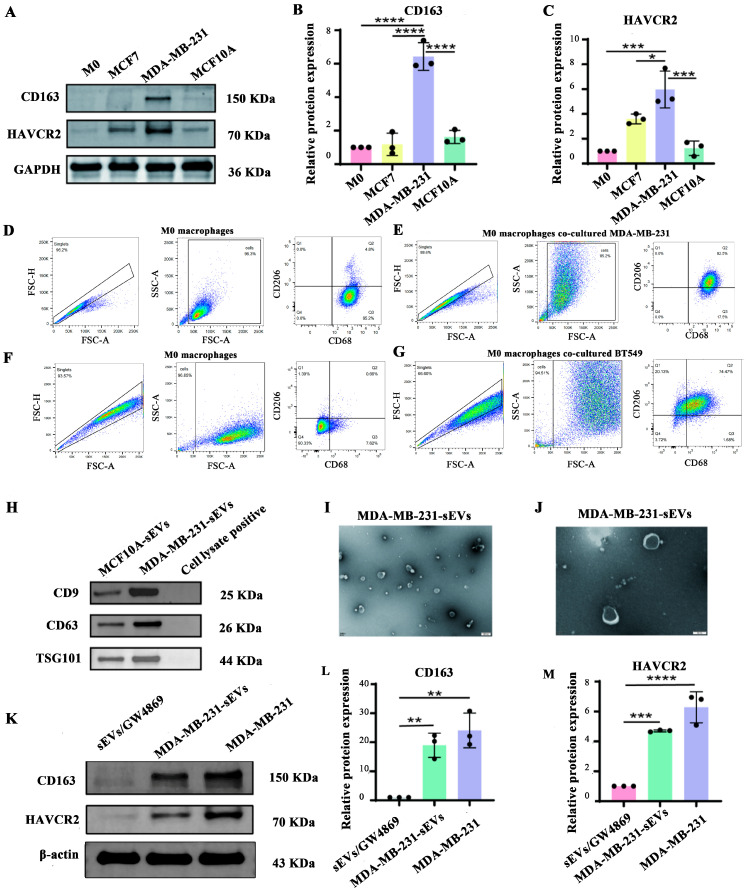
TNBC-derived sEVs are associated with macrophage upregulation of M2-associated markers and HAVCR2 upregulation (indirect GW4869 evidence; no direct causal link established). (**A**–**C**) Western blot analysis revealed that after different types of breast cancer cells were cocultured with macrophages, the expression of HAVCR2 and the M2-associated marker CD163 in macrophages was upregulated. (**D**–**G**) Flow cytometry revealed that the M2 marker CD206 was significantly elevated in macrophages after TNBC cells were cocultured with macrophages. (**H**–**J**) The sEVs of the collected TNBC cells were identified via electron microscopy and Western blotting. (**I**): Scale bars, 200 nm. (**J**): Scale bars, 100 nm. (**K**–**M**) WB results showing that when MDA-MB-231 cells were cocultured with macrophages treated with the sEV generation inhibitor GW4869, the levels of HAVCR2 and the M2 marker CD163 were decreased. All data were analyzed by using unpaired Student’s *t*-tests and are shown as the means ± SD. *p < 0.05; **p < 0.01; ***p < 0.001; ****p < 0.0001.

To study the polarization effect of breast cancer cells on macrophages *in vitro*, different types of breast cancer cells were cocultured with THP-1-derived M0 macrophages. After coculture, morphological analysis revealed that the macrophages were irregularly shaped and exhibited long pseudopods. In addition, the expression of HAVCR2 and the M2-associated marker CD163 was upregulated in cocultured macrophages according to WB (Fig. 6A–C), especially in the TNBC cell line MDA-MB-231. These findings indicate that TNBC cells are correlated with elevated expression of M2-associated markers in macrophages. Moreover, the results of flow cytometry also revealed that the M2-like associated marker CD206 was significantly increased in the co-culture group (Fig. 6D–G), which is associated with HAVCR2 upregulation. Morphology, WB and flow cytometry revealed that TNBC cells induce upregulation of M2-associated markers and HAVCR2 in macrophages. These effects are hypothesized to be affected by TNBC-derived sEVs, as validated by GW4869 inhibition.

### 3.8 TNBC-Derived sEVs Are Associated with Macrophage Upregulation of M2-Associated Markers and HAVCR2 Upregulation

To evaluate the potential involvement of sEVs in regulating macrophages, TNBC cell-derived sEVs were isolated and validated. sEVs from TNBC cells were collected and identified by electron microscopy and WB (Fig. 6H–J). WB showed that co-culture with MDA-MB-231 cells in the presence of the sEV biogenesis inhibitor GW4869 was associated with reduced levels of HAVCR2 and the M2 marker CD163 in macrophages (Fig. 6K–M). Treatment with GW4869, an inhibitor of sEV biogenesis, significantly reduced the expression of CD163 and HAVCR2 in macrophages co-cultured with MDA-MB-231 cells. While these results support the notion that TNBC-derived sEVs are correlated with M2 polarization and HAVCR2 expression, the potential off-target effects of GW4869 suggest that a direct causal link cannot be definitively established without rescue experiments. Therefore, these findings should be interpreted as supportive evidence for the involvement of sEVs in this process.

### 3.9 Macrophages Treated with sEVs from MDA-MB-231 Cells Are Associated with Increased Migration and Invasion of TNBC Cells

**Figure 7:**
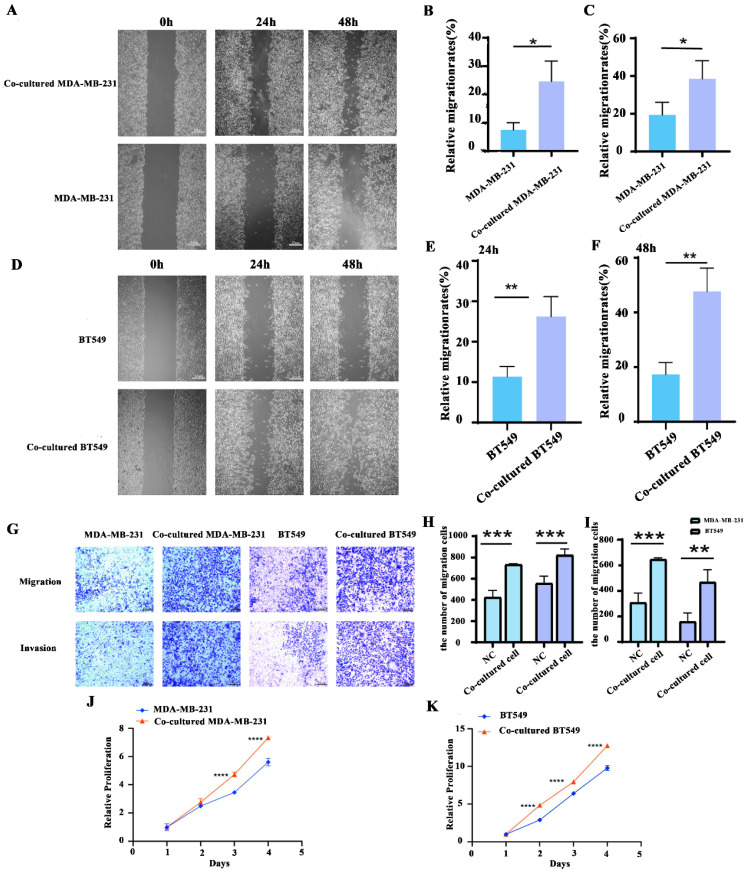
Macrophage exposure to TNBC-derived sEVs correlates with enhanced migratory and invasive capacities of TNBC cells (supporting the sEV paracrine hypothesis; direct sEV-related regulation remains unconfirmed). (**A**–**F**) Scratch tests revealed that TNBC cell invasion was promoted upon cocultured with sEV inhibitor-treated macrophages. Scale bars, 50 μm in (**A**,**D**). (**G**–**I**) Transwell assays revealed that cocultured with sEV inhibitor-treated macrophages promoted the proliferation and invasion of TNBC cells. Scale bars, 50 μm. (**J**,**K**) CCK-8 assays also revealed that TNBC cell proliferation was promoted following cocultured with sEV inhibitor-treated macrophages. All data were analyzed by using unpaired Student’s *t*-tests and are shown as the means ± SD. *p < 0.05; **p < 0.01; ***p < 0.001; ****p < 0.0001.

We further co-cultured sEV inhibitor-pretreated macrophages with MDA-MB-231 or BT549 TNBC cells. The results revealed that macrophages receiving sEVs from TNBC cells exhibited increased migratory and invasive capabilities (Fig. 7A–I). Moreover, the results of the CCK-8 assay also indicated that the proliferation capabilities of the MDA-MB-231 and BT549 cells were increased after coculture (Fig. 7J,K). These findings indicate that macrophages treated with TNBC-derived sEVs and polarized toward an M2-like phenotype are correlated with enhanced proliferative and progressive phenotypes in TNBC cells, supporting the existence of sEV-related paracrine crosstalk between macrophages and TNBC cells in the TME.

## 4 Discussion

Breast cancer is considered the most common cancer type and the leading cause of cancer-related death among women. TNBC is one of the most intractable and lethal malignancies and accounts for 24% of newly diagnosed cases of breast cancer [34]. The characteristic lack of expression of ER, PR and HER2 in TNBC limits the use of common therapies, such as conventional endocrine therapy [35]. Thus, alternative therapeutics are needed to improve outcomes for patients with TNBC. The successful application of immune checkpoint inhibitors (ICIs) has inaugurated the immunotherapy era for TNBC treatment. However, recent findings indicate the limited efficacy of PD-L1 and PD-L1 inhibitor monotherapy, and only a small proportion of patients can benefit from this approach. Based on the fundamental principles of immunotherapy and the characteristics of the TNBC TME, the search for novel immune checkpoint targets is expected to further improve treatment efficacy and expand the population of patients who benefit from immunotherapy [36]. 

Recently, studies have shown that functional communication between tumour cells and immune cells in the TME is critical for regulating signalling pathways that promote tumour initiation and progression. The use of single-cell transcriptomic technology across human cancers provides an unprecedented opportunity to explore the TME in greater depth [37,38,39]. The present study utilized public scRNA-seq datasets of primary tumour tissue from 13 TNBC patients [33], encompassing nearly 60,000 cells and four TNBC subtypes. Consistent with previous studies, our integrated analysis provided evidence that TNBC patients have relatively higher proportions of immune cells than patients with other breast cancer subtypes [40,41,42]. An improved understanding of the interplay between TNBC tumour cells and their TME supports the adoption of a new view of TNBC as an ecosystem [43]. Accordingly, immunotherapeutic strategies targeting TME immune cells hold great potential for improving clinical benefits in TNBC patients.

One of the challenges in cancer treatment is related to tumour-infiltrating cells, particularly immune cells associated with tumour progression. Among them, tumour-associated macrophages are the most abundant immune cells in many cancer types. These macrophages contribute to the formation of a TME that promotes immunosuppression, metastasis/invasion, angiogenesis and drug resistance [44]. Some tumour-associated macrophage states are associated with the response to treatment or the lack thereof in breast cancer [43,45,46,47]. In this study, macrophages in the TME were observed to be involved in more cell–cell interactions than other cell types in the HER2 and luminal B subtypes and particularly in the TNBC subtype. ICs, as the mainstay of antitumour immunotherapy, is considered promising for TNBC treatment [48]. The key role of IC inhibitors in the treatment of TNBC is being defined. Different combination strategies provide additional insights into improving the efficacy of immunotherapy in TNBC patients [36]. However, early-phase trials with monoclonal antibodies against PD-L1 have shown very limited responses in TNBC. Thus, it is necessary to identify other valuable ICs in TNBC. 

HAVCR2 (also known as TIM-3) is an immunoregulatory protein that is expressed in various immune cells [49,50,51]. Tim-3 has been recognized as a promising immune checkpoint target for tumour immunotherapy [52]. The expression of HAVCR2 in a wide range of neoplasms has been a trending topic in recent years. The analysis of HAVCR2 expression in TCGA dataset revealed its broad expression in different types of cancer. A growing body of recent evidence indicates that its differential expression in various tumour types is related to tumour prognosis [53]. As a critical immune checkpoint post PD-1/PD-L1 and CTLA-4, HAVCR2/TIM-3 has multiple targeted inhibitors (biological/chemical agents) in preclinical and clinical development [54]. Several HAVCR2-targeted antibodies are in early clinical trials. Sabatolimab (MBG453, Novartis) combined with hypomethylating agents achieves a 56.9% objective response rate (ORR) in high-risk MDS patients [55]. Cobolimab (TSR-022) shows manageable safety in PD-1/PD-L1-refractory solid tumours, with synergistic efficacy when combined with PD-1 inhibitors [55]. Hengrui Medicine’s SHR-1702 is also in Phase I trials for MDS and solid tumours [56]. Therefore, HAVCR2 has the potential to serve as a prognostic marker and valuable candidate molecule for therapeutic research in TNBC.

HAVCR2 has been reported to be associated with the regulation of immune responses in autoimmunity and tumours [50,57,58,59]. These reports are consistent with the results of this study. According to our analysis, HAVCR2 is expressed mainly in the macrophages of breast cancer tissues, regardless of subtype. Tumour-associated macrophages can simultaneously display both classically activated proinflammatory M1 (antitumour) and alternatively activated anti-inflammatory M2 (tumour-promoting) phenotypes [16]. Interestingly, our analysis and experiments revealed that HAVCR2 expression is upregulated in macrophages with upregulated M2-associated markers and associated with poor clinical outcomes. The sEVs secreted by TNBC cells are associated with the upregulation of HAVCR2 in M2 TAMs, and this concurrent change is correlated with the invasion and metastasis of TNBC cells. These results suggest that future investigation may explore whether anti-HAVCR2 antagonistic antibodies could modulate HAVCR2+ M2-like macrophage function and antitumour immunity in TNBCs. However, it is not clear how sEVs in tumours promote macrophage polarization and HAVCR2 expression. Our subsequent research will further explore this mechanism.

Notably, the present study has some main limitations that need to be addressed in future research. First, we only confirmed the functional role of TNBC-derived sEVs in regulating macrophage M2 polarization and HAVCR2 expression, but did not characterize the specific cargo (e.g., miRNAs, proteins, lipids, circRNAs) in these sEVs that mediates the above biological effects. sEVs exert intercellular communication functions mainly through their encapsulated bioactive molecules, and identifying the key functional cargo is the core to elucidate the molecular mechanism of sEVs regulating macrophage phenotype and immune checkpoint expression. Second, it remains unclear whether elevated HAVCR2 expression in macrophages is a direct effect of TNBC-derived sEVs or a secondary consequence of sEV-associated M2 polarization. HAVCR2 is a marker of immune cell exhaustion, and its expression may be closely linked to the M2 polarization process of macrophages, but the causal relationship between the two has not been verified in this study. Third, a notable limitation of this study is the exclusive detection of M2-associated markers in *in vitro* sEV-associated macrophage polarization assays, without parallel M1 marker quantification or standard M1/M2 positive controls (LPS/IFNγ for M1; IL-4/IL-13 for M2). The intrinsic plasticity of PMA-differentiated THP-1 macrophages means non-specific activation cannot be fully excluded. However, scRNA-seq reanalysis of TNBC TAMs confirmed suppressed M1 marker transcription, indirectly supporting sEV-associated polarization is M2-dominant. Subsequent studies will add M1/M2 positive controls and parallel marker detection (mRNA/protein/cytokine) to verify selective M2 skewing. Fourth, evidence supporting sEV-associated regulation of macrophage polarization relies solely on GW4869-related inhibition of sEV biogenesis. While GW4869 is a commonly used tool to block sEV release, it may have off-target effects on cellular processes unrelated to sEV biogenesis. A rescue experiment, in which purified sEVs are added back to GW4869-treated cultures, would be necessary to definitively confirm the specific role of sEVs. Future studies will incorporate this rescue experiment to strengthen the causal conclusion. Fifth, all analyses based on TCGA data rely on bulk RNA-seq, which only provides tissue-level transcriptomic information and cannot define or identify discrete HAVCR2+ M2 macrophage cell subsets at the single-cell level. The observed correlation between HAVCR2 expression and M2-like macrophage signatures merely reflects transcriptional co-occurrence at the tissue level, and cannot directly confirm the expression of HAVCR2 on macrophages with upregulated M2-associated markers-this conclusion was instead validated by single-cell RNA-sequencing, snRNA-seq and immunofluorescence staining at the single-cell/tissue level. Future studies will combine single-cell spatial transcriptomics to further confirm the spatial localization and cell-specific expression of HAVCR2 in the TNBC microenvironment. Sixth, the characterization of macrophage molecular changes in this study relies solely on a limited set of M2-associated markers (CD163, CD206, MRC1) and morphological observations, without including additional M2-associated markers (e.g., IL-10, TGF-β, CD204) or functional readouts (e.g., immunosuppressive cytokine secretion, antigen presentation capacity) to validate a comprehensive macrophage program switch. All conclusions are therefore strictly restricted to the upregulation of M2-associated markers (an objective experimental observation), and no inferences of complete M2 polarization or functional reprogramming are made. Future studies will expand the marker panel and perform functional assays to characterize macrophage immunosuppressive activity and antigen presentation capacity, addressing this limitation.

## 5 Conclusions

We performed a comprehensive single-cell level analysis of breast cancer subtypes and identified a specific HAVCR2+ M2 macrophage subgroup in the TNBC microenvironment by scRNA-seq and snRNA-seq, with in-house snRNA-seq data (three TNBC patients) providing supportive evidence consistent with public scRNA-seq dataset findings; notably, the proportion of HAVCR2+ M2 macrophage can predict the prognosis of TNBC. This TAM subgroup is correlated with tumour progression and metastasis. These observations generate the hypothesis that targeting TNBC-derived sEVs or HAVCR2 in TAMs merits further preclinical evaluation as potential strategies for TNBC investigation. Hence, targeting HAVCR2 in TAMs is proposed as a potential novel strategy for TNBC therapy, and inhibiting the secretion of TNBC sEVs (a hypothetical mediator of macrophage reprogramming) may represent a complementary approach for sensitizing anti-HAVCR2 therapy, both of which require direct validation of sEV function and *in vivo* testing.

## Data Availability

Not applicable.
